# Alterations of presynaptic proteins in autism spectrum disorder

**DOI:** 10.3389/fnmol.2022.1062878

**Published:** 2022-11-17

**Authors:** Xin Yi Yeo, Yi Tang Lim, Woo Ri Chae, Chungwon Park, Hyokeun Park, Sangyong Jung

**Affiliations:** ^1^Institute of Molecular and Cell Biology, Agency for Science, Technology and Research, Singapore, Singapore; ^2^Department of Psychological Medicine, Yong Loo Lin School of Medicine, National University of Singapore, Singapore, Singapore; ^3^Department of BioNano Technology, Gachon University, Seongnam, South Korea; ^4^Division of Life Science, The Hong Kong University of Science and Technology, Kowloon, Hong Kong SAR, China; ^5^Department of Physics, The Hong Kong University of Science and Technology, Kowloon, Hong Kong SAR, China; ^6^State Key Laboratory of Molecular Neuroscience, The Hong Kong University of Science and Technology, Kowloon, Hong Kong SAR, China; ^7^Department of Physiology, Yong Loo Lin School of Medicine, National University of Singapore, Singapore, Singapore

**Keywords:** presynaptic proteins, synaptopathy, presynaptic vesicle dynamics, vesicle release machinery, synaptogenesis, autism spectrum disorders (ASD)

## Abstract

The expanded use of hypothesis-free gene analysis methods in autism research has significantly increased the number of genetic risk factors associated with the pathogenesis of autism. A further examination of the implicated genes directly revealed the involvement in processes pertinent to neuronal differentiation, development, and function, with a predominant contribution from the regulators of synaptic function. Despite the importance of presynaptic function in synaptic transmission, the regulation of neuronal network activity, and the final behavioral output, there is a relative lack of understanding of the presynaptic contribution to the pathology of autism. Here, we will review the close association among autism-related mutations, autism spectrum disorders (ASD) phenotypes, and the altered presynaptic protein functions through a systematic examination of the presynaptic risk genes relating to the critical stages of synaptogenesis and neurotransmission.

## Introduction

When first described, autism was regarded as a neuropsychiatric condition rooted in psychosocial distress and family burden and characterized by a range of emotional and communication defects from the early stages of life (Kanner, 1968; MacCULLOCH and Sambrooks, 1973). Subsequently, patients with metabolic defects such as phenylketonuria or creatine deficiency syndromes (Manzi et al., 2008) and mitochondrial disorders were observed to suffer similar autistic symptoms (Frye, 2020). Autistic patients often have problems with social communication, interactions, and attention and consequently present with abnormal behaviors (Fakhoury, 2015). Due to the lack of biological understanding of the development of autism and the wide variation in severity of symptoms, autism has been considered a spectrum disorder (ASD), with diagnosis hinged on the clinical phenotypes observed in autistic individuals reported in the Diagnostic and Statistical Manual of Mental Disorders (DSM) (Rosen et al., 2021).

Follow-up epidemiological studies further revealed the involvement of intellectual disability in autistic individuals (Kerbeshian et al., 2008), high adjusted concordance rate of autism among monozygotic twins (60%) and siblings (2%) compared to general population prevalence rates of 0.04% (Folstein and Rutter, 1977; Bolton et al., 1994; Bailey et al., 1995), and a skewed male preference for the development of ASD (Smalley, 1988), suggesting a genetic involvement in etiology of ASD. In an attempt to pinpoint the etiology of ASD, karyotypic and linkage studies have presented the debatable association of susceptibility loci on chromosome 2q (Buxbaum et al., 2001), 7q (International Molecular Genetic Study of Autism Consortium, 1998), 15q (Shao et al., 2003), 16p, and 19 (Liu et al., 2001) with ASD development. Though not all candidates identified in these first genetic studies are convincingly associated with the core symptoms of ASD (Talebizadeh, 2002; Zhang et al., 2002), genes with validated links with ASD phenotypes, such as the contactin-associated protein-like 2 (CNTNAP2) (Alarcón et al., 2008), gamma-aminobutyric acid type A receptor gamma 3 subunit (GABRG3) (Buxbaum et al., 2002), methyl-CpG binding protein 2 (MeCP2) (Lam, 2000; Carney et al., 2003), ubiquitin-protein ligase E3A (UBE3A) (Nurmi et al., 2001), and neuroligin 3 (NLGN3) (Paris Autism Research International Sibpair Study et al., 2003) point to the neurodevelopmental and multifactorial nature of autism.

More importantly, it is clear that autistic phenotypes likely stem from the disruption of processes critical for neuronal differentiation, development, and function. Interestingly, the re-examination of the genetic landscape of autistic individuals with new hypothesis-free whole genome sequencing and specific single nucleotide polymorphisms (SNP) identification methods have resulted in the confirmation and preferential pick-up of a myriad of highly penetrant mutations and variations in regulators of synaptic function (Buxbaum et al., 2007; Zoghbi and Bear, 2012; GiovedÃ et al., 2014; Leblond et al., 2014). Synapses are the basic computational unit of the nervous system responsible for the organized transmission and processing of information in the central nervous system (Juusola et al., 1996), which inevitably modulates an individual’s behavioral output and cognitive abilities (Woodburn et al., 2021). As the synaptic function is dependent on a series of coordinated and organized processes that drives synapse formation (Naskar et al., 2019), maintenance, and activity-dependent neurotransmitter release (Südhof and Malenka, 2008), it is not surprising that mutations in synaptic regulators results in a predisposition to the development of cognitive deficits.

**FIGURE 1:**
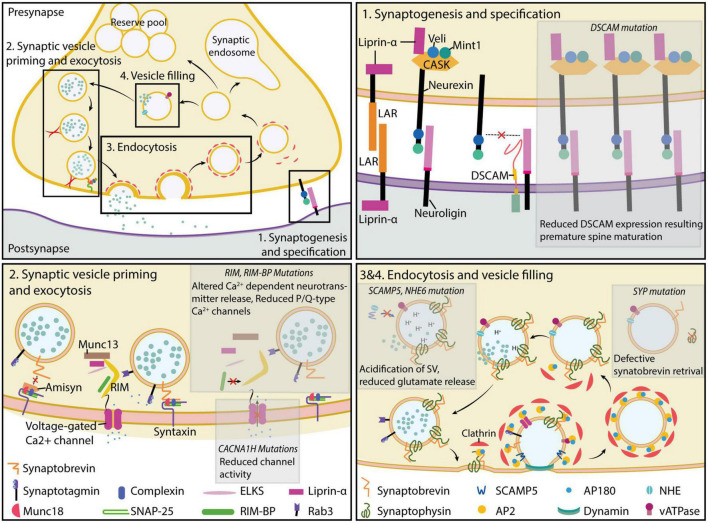
Molecular players of presynaptic function. Presynaptic formation and function are critically dependent on the expression and localization of proteins involved in the process of synaptogenesis and specification **(top right)** (section “Synaptogenesis and specification”), synaptic vesicle priming and exocytosis **(bottom left)** (sections “Synaptic vesicle priming” and “Synaptic vesicle exocytosis”), retrieval of synaptic vesicles **(bottom right)** (section “Retrieval of vesicle from plasma membrane and endocytosis”), and filling of empty synaptic vesicles **(bottom left)** (section “Regulation of synaptic vesicle filling and synaptic vesicle pools”). Mutations disrupting presynaptic protein function impair normal synaptic formation and neurotransmission, resulting in a loss of synaptic function. Further discussion of the mutations and their potential implications in autism etiology can be found in the main text.

There has been a preferential focus on the factors involved in the organization of synaptic structure and functional outcome that makes up a significant proportion of the risk genes identified in the autistic population (Guang et al., 2018). The extensive investigation of the contribution of the neuroligin (NLGN)/neurexin (NRXN) family of cell adhesion molecule and SRC Homology 3 Domain (SH3) and multiple ankyrin repeat domains (SHANK) family of glutamatergic postsynaptic density protein and their autism-associated mutations (Jiang and Ehlers, 2013; Trobiani et al., 2020), which cumulate into the excitation-inhibition balance model of autism development (Sohal and Rubenstein, 2019). Despite the potential involvement of presynaptic active zone proteins in neuronal circuit regulation throughout various stages of development and the importance of presynaptic neurotransmitter release machinery in basic neuronal signal propagation, there has been a lack of understanding of the presynaptic contribution to the development of ASD. In this review, we summarize the reported autism-related mutations related to presynaptic functionality and their potential impact on the development of autistic phenotypes (Table 1). We also recognize the previous attempt to understand the state of involvement of the presynaptic vesicle release machinery in general neurodevelopmental disorders (Bonnycastle et al., 2021). Nevertheless, by examining the novel presynaptic autism risk factors, we aimed to provide a renewed and balanced perspective of the current understanding of the presynaptic involvement in cognitive defects (Figure 1), which are characteristic of autism pathogenesis.

**TABLE 1:** Autism-associated mutations in presynaptic proteins.

Protein	Gene Symbol	SFARI Score	Autism-related mutations	Function/biomolecular observation	Clinical phenotype	References
**Presynaptic organization**						
Down syndrome cell adhesion molecule immunoglobulin superfamily/ Chromodomain Helicase DNA Binding Protein 2	DSCAM/CHD2	1	c.2051del(T)	Lower protein expression levels, decrease in axonal length, reduction in NR1 (a subunit of NMDA-R) density and NMDA-R currents	Behavioral disorders, sleeping, and communication disorders	Chen et al., 2022
			p.Pro356Leufs*5	Impact of specific mutation unknown. DSCAM dysfunction results in impairment of axon extension and guidance in neurodevelopment, which could be manifested in these particular mutations within DSCAM	Developmental delay in speech, repetitive and obsessive behavior, hyperactivity, and attentional problems	Gandawijaya et al., 2020; Hamdan et al., 2009
			p.Arg1685His		Moderate to severe intellectual disability, developmental delay in speech, sleep problems, GI disturbances, repetitive and obsessive behavior, hyperactivity and attentional problems, anxiety, aggressive behavior	Carvill et al., 2014; Gandawijaya et al., 2020
Liprin-α	PPFIA1	2	Breakpoint in intron 8 of 11q13.3	Impact of specific mutation unknown. Mutation in Liprin-a1 protein resulted in the impairment of activity-dependent degradation of liprin-a1. This results in an inhibition of dendritic morphogenesis and reduction in synaptic density. The dendritic targeting of LAR is also impaired	Speech delay, moderate intellectual deficiency, facial dysmorphism, autistic behavior	Butz et al., 1998; Moog et al., 2015
Calcium/calmodulin dependent serine protein kinase	CASK	1	p.Ser475Ile	Reduction in synapses per neuromuscular junction, reduces synaptic vesicle recycling, sEJC frequency, and evoked neurotransmitter release	Microcephaly, developmental delay in speech and walking, poor verbal communication, impairment in social communication, repetitive behavior	Fowler et al., 2017; Schluth-Bolard et al., 2013
**Synaptic vesicle exocytosis**						
Ca^2+–^dependent secretion activator	CAPS/CADPS	2	CADPS2 (Δexon3)	Impaired translocation of CADPS2 to axon terminals	Predisposition to autism	Imig et al., 2014
			pVal1137Met in CADPS2	Impact of specific mutation unknown. CADPS-KO resulted in reduction in release of neuropeptide oxytocin into plasma from the pituitary gland	Motor clumsiness, epilepsy, mild intellectual disability, and mild language developmental delay	Okamoto and Südhof, 1997; Südhof, 2013
			p. Asp1113Asn in CADPS2	Disrupted its interaction with dopamine receptor type 2	Social withdrawal at a young age, repetitive play, attention deficit and learning difficulties, irregular sleep-wake rhythm, mild cognitive impairment	
Rab3A-interacting molecule	RIM/RIMS	1	Insertion of nucleotide A at amino acid position 196 of protein (13162.p1)	Impact of these specific mutations is unknown. Knockout of RIM results in impairment of neurotransmitter release, alteration of Ca2+ dependence of neurotransmitter release, reduction in presynaptic P/Q-type Ca2+ channel levels	Anxiety, depression, withdrawn, slightly lower IQ	Bucan et al., 2009; Dong et al., 2014
			13497.p1			Bucan et al., 2009; Südhof, 2012
Rab3-interacting molecule-binding protein	RIM-BP/TSP0AP1	2	Exonic deletions in regions containing the Src homology-3 and fibronectin, type III domains	Impact of various exonic deletions unknown. RIM-BP acts as a binding partner of RIM via RIM-BP’s SH3 domain. It can be postulated that the deletions in the SH3 domain results in impairment of binding of RIM to RIM-BP	Phenotype not reported	Iossifov et al., 2012; Jacquemont et al., 2014
Synapsin	SYN	1	Q555X	DE-domain binding to SV, interaction with all SH3 domains (except PLCγ) is abolished. Reduced phosphorylation by CaMKII and MAPK/ERK. Impaired axon elongation and release of reserve pool and readily releasable pool	Diagnosed with ASD according to ADI-R and ADOC-G, exhibit idiopathic partial epilepsy	Corradi et al., 2014
			A550T	Impairment of presynaptic localization of synapsin		
			T567A			
			A51G	Impact of specific mutation not reported. Syn1 knockout shows an impairment in the size and trafficking of synaptic vesicle pools		
			W356X	Potentially cause a defect in SV trafficking and neurotransmitter release	Impairments in social interaction, use of language, restricted and repetitive behavior, outbursts of severe aggression	Garcia, 2004
	SYN2	2	A94fs199X	Not expressed in mutational studies in HeLa cells or primary neurons	Diagnosed with ASD according to ASQ, ADOS-G and ADI-R	Haas and DeGennaro, 1988
			Y236S	Impairment of of RP size and total synaptic vesicle content	Diagnosed with ASD according to ASQ, ADOS-G and ADI-R, ASD, higher functioning	
			G464R	Impairment of axonal outgrowth and dendritic branching	Diagnosed with ASD according to ASQ, ADOS-G and ADI-R.	
Voltage-gated calcium channel	CACNA1C/Ca_v_1.2	1	G406R	Aberrant calcium signaling	Timothy syndrome, language deficit, impairment in social development	Staras et al., 2010
	CACNA1H/Ca_v_3.2	2	R212C, R902W, W962C, R1817Q/A1974V	Alteration of channel kinetics and voltage-dependent gating properties	Diagnosed with ASD	Splawski et al., 2006
Synaptotagmin	SYT	S	I368T	Reduction in rate of SV exocytosis, acceleration in SV endocytosis	Early onset mixed hyperkinetic movement, severe motor delay and profound cognitive impairment	Baker et al., 2018
			M303K	Low levels of expression of protein, and retention at nerve terminals	Esotropia, infant hypotonia, ataxia, angry outbursts, impatience, impulsivity	Kumar et al., 2009
			D304G	Diffuse localization post-neuronal stimulation, reduced rate of exocytosis	Progressive contractures, scoliosis, gastro-esophageal reflux, strabismus hypermetropia, infant hypotonia, stereotypies, repeated aggressive behavior	
			D366E	Diffuse localization post-neuronal stimulation, reduced rate of exocytosis	Laryngomalacia, atrial septal defect, lumbar lordosis, valgus deformities, sleep and central apnea, constipation, esotropia, nystagmus and strabismus, infant hypotonia, object mouthing, head banging, bites and scratches self when frustrated, hand-biting, screaming, obsessions and repetition, hand-chewing	
			N371K	Reduced rate of exocytosis	Dermoid cysts, feeding difficulties, gastrointestinal problems, sleep apnea, nystagmus, infant hypotonia, dystonia, dyskinetic cerebral palsy, trunk and limb dystonia, chorea, screaming episodes, teeth grinding, hand-chewing	
Double C2-like domain-containing protein	DOC2A	Not Listed	77883G > A	Impact of specific mutation unknown. Predicted to alter transcription factor binding sites for several brain-expressed genes	Diagnosed with ASD	Chernomordik et al., 1987; Sakaguchi et al., 1999
			M225I	Alterations in synaptic transmission and reduction in long term potentiation		
Amisyn	STXBP6	Not Listed	Inverted duplication of proximal chromosome 14	Thought to be a negative modulator of SNARE-dependent vesicle priming	Speculated involvement in ASD	Groffen et al., 2006; Koopmans et al., 2018; Tran et al., 2020
**Synaptic vesicle endocytosis**						
Dual Specificity Tyrosine Phosphorylation Regulated Kinase 1A	DYRK1A	1	p.Ile48Lysfs*2	Impact of specific mutations unknown. In Dyrk1A^±^ mice, cell counts showed increased neuronal densities in some brain regions and a specific decrease in the number of neurons in the superior colliculus. There were also decrease in sizes of stratum griseum superficiale and stratum opticum, likely due to reduction in neuronal numbers	Intellectual disability, speech and motor difficulties, microcephaly, feeding difficulties, and vision abnormalities	Sadasivam and DeCaprio, 2013
			p.Ala498Profs*61			
			p.Lys406Argfs*44			
			p.Arg255*			
			p.Ile468Aspfs*17			
			p.Asn151Lysfs*12			
			p.Leu295Phe			
			p.Lys416Asnfs*35			
			Deletion of 1 nucleotide in amino acid 487		Developmental delay, behavioral problems, impaired speech and lower cognitive ability	Ma et al., 2011
			p.Glu153*		Developmental delay in speech, repetitive behavior, microcephaly, sleep problems, GI disturbances, hyperactive behavior, febrile seizures, C-section, premature birth, neonatal and childhood feeding problems	Wang et al., 2016
			p.Gln201*		Phenotype not reported	Satterstrom et al., 2020
**Synaptic vesicle trafficking**						
Synaptophysin	SYP	3	p.Ala84Gly	Impact of this specific mutation unknown. Knockout studies show a defective synaptobrevin II retrieval, and slowing of synaptic vesicle (SV) endocytosis. However, there is no effect on the overall turnover rate of SV	Phenotype not reported	Sadasivam and DeCaprio, 2013; Satterstrom et al., 2020
**Synaptic vesicle filling**						
Na + /H + exchanger 6	SLC9A6/NHE6	1	p.H171fs	Impact of specific mutations unknown. Involved in the acidification of SV. Knockout mice present an impairment of endosomal maturation and trafficking	Microcephaly, developmental delay, verbal language absent, epilepsy	Gilfillan et al., 2008; Lee et al., 2021
			p.R468X		Developmental delay, verbal language absent, epilepsy, sleep disturbance	
			p.V144_R169 del		Microcephaly, developmental delay, verbal language absent, epilepsy	
Na + /H + exchanger 9	SLC9A9/NHE9	2	S438P	Impact of specific mutations unknown. NHE9 plays a role in glutamate reuptake in astrocytes. Its impairment results in the increase in synaptic glutamate and neuronal hyperexcitability	Phenotype not reported	Cardon et al., 2016
			L236S			
			V176I			
Secretory carrier-associated membrane protein	SCAMP5	Not Listed	breakpoint on chromosome 15q	Impact of specific mutation unknown. Knockdown of SCAMP5 result in the inhibition of axonal trafficking and presynaptic localization of NHE6, leading to hyper acidification of the SVs and a reduction of the quantal size of glutamate release	Mildly delayed early psychomotor development, markedly delayed language and social development, pronounced ritualistic behavior and stereotyped body movements, mood changes, anxiety, episodic aggression and auto-mutilation	Hubert et al., 2020; Rosen et al., 2018

SFARI Gene is a comprehensive autism risk gene database that includes all genes with some form of correlation to ASD pathophysiology. Genes listed in the database are scored based on the strength of evidence of gene linkage with autism. The SFARI Gene scoring categories are as follows: S, mutations associated with a substantial risk of ASD development but may be lead to characteristics not specific to ASD; 1, genes directly implicated in ASD; 2, genes with two reported *de novo* likely-gene-disrupting mutations and identified by gene-wide association study accompanied by evidence of functional effect related to ASD; 3, unvalidated genes with single reported *de novo* likely-gene-disrupting mutation and evidence from association study; Not Listed, a gene not currently listed in the database. The symbol * is used in nucleotide numbering and to indicate a translation termination (stop) codon for gene variations.

## Developmental profile of presynaptic terminals and their involvement in neuronal function

### Synaptogenesis and specification

Synapses are specialized and asymmetrical structures formed through the initial formation of dendritic or axonal filopodia to bring nascent presynaptic sites closer to targeted postsynaptic specializations. In the neuron, the actin-capping protein (CP) works in concert with actin-related protein 2/3 (Arp2/3) to prevent further elongation of filopodia structures (Akin and Mullins, 2008; Fan et al., 2011), likely promoting the formation of more stable, putative synaptic structures. Subsequently, the anti-capping protein-enabled/vasodilator-stimulated phosphoprotein (Ena/VASP) regulates presynaptic actin polymerization, which affects synaptic protein anchoring and the eventual bouton size (Lin et al., 2010). The process is followed by the recruitment of cell adhesion molecules (CAMs) to stabilize axo-dendritic contact. The importance of CAMs in synapse formation, specialization, and function is well understood and has been extensively examined in other reviews (Dalva et al., 2007; Kilinc, 2018; Südhof, 2018). Previous reviews on the CAMs’ ASD-associated mutations and their link to autism development have provided many insights into their roles in ASD pathology (Ye et al., 2010; Redies et al., 2012; Gandawijaya et al., 2020; Tromp et al., 2021).

The DS Cell Adhesion Molecule (DSCAM), also known as chromodomain helicase DNA binding protein 2 (CHD2), is a member of the neuronal immunoglobulin superfamily of molecules broadly expressed in the nervous system and implicated in the process of pathfinding (Liu et al., 2009), axon branching (Wang et al., 2002), and dendritic arborization (Maynard and Stein, 2012). The gene coding for DSCAM has been mapped to chromosome 21q22.12 → q22.3, commonly duplicated in Down syndrome patients (Yamakawa, 1998), and its upregulation has been linked to altered neuronal circuits and learning and behavioral abnormalities in early studies on the animal models of Down syndrome (Sago et al., 1998). In addition to its link with Down syndrome, DSCAM is a strong ASD risk gene recurringly identified in various genome association studies (Iossifov et al., 2014; Sanders et al., 2015; Wang et al., 2016). Mutations that result in the premature termination of DSCAM expression have also been identified within the human autistic population, supporting the importance of DSCAM function in general neurological function.

DSCAM exhibits prominent synaptogenic capabilities. In *Drosophila* and Aplysia, DSCAM is involved in the regulation of synapse targeting (Millard et al., 2010) and the activity-dependent clustering of α-amino-3-hydroxy-5-methyl-4-isoxazolepropionic acid (AMPA) receptors (Li et al., 2009), leading to an alteration in presynaptic size (Kim et al., 2013). This observation is likely due to the preferential interaction with, and aggregation of, postsynaptic proteins associated with nanoclusters of protein determinants of synapse maturity and functionality, similar to effects observed with neurexin ligands (Nozawa et al., 2022). Furthermore, DSCAM can negatively regulate synaptic maturation through competitive interaction with neuroligin 1 (NLGN1) and the inhibition of NLGN1-NRXN1β-mediated synaptic specification (Chen et al., 2022) in the developing mouse cortex. Using an induced pluripotent stem cell (iPSC)-derived model of telencephalic neurons, an early truncated variant of DCAM (amino acid 684) resulted in a reduced DSCAM/N-methyl-D-aspartate receptor subunit 1 (NR1) colocalization. As a result, the synaptic N-methyl-D-aspartate (NMDA) receptor response was compromised (Lim et al., 2021), suggesting DSCAM’s role in synaptic maintenance.

CASK is an alternative presynaptic scaffold protein encoded by chromosome Xp11.4. Due to its localization on the X chromosome, pathogenic variants are disproportionately identified in the female-dominant X-linked microcephaly with pontine and cerebellar hypoplasia (OMIM 300749) and FG syndrome 4 (OMIM 300422) (Piluso et al., 2009; Moog et al., 2015), with a comparatively rare occurrence in general autistic patients (Gupta et al., 2014; Seto et al., 2017). Identified mutations are widely distributed along the gene without preferential mutation spots (Moog et al., 2015), while observed phenotypes vary in individuals. Hence, little can be deduced regarding the dominance of mutations and the key impact of CASK on the development of intellectual disorders and ASD. In normal physiology, CASK is a neurexin-binding protein (Hata et al., 1996) with a suspected role as a synaptic organizer that links cell adhesion and transsynaptic signaling with vesicle exocytosis (Butz et al., 1998). Despite an absence of effect on neuron excitability, and microstructure of synapses when CASK is ablated (Atasoy et al., 2007), there is a reduction in spontaneous synaptic events and synaptic vesicle cycling in the *Drosophila* neuromuscular junction (Chen and Featherstone, 2011) that corroborate the potential function of CASK in presynaptic vesicle exocytosis. Further work is required to pinpoint the precise neurological function of CASK and its correlation with the observed neurodevelopmental conditions.

The ability of non-neuronal HEK293 cells artificially expressing postsynaptic-specific CAM to induce the formation of presynaptic specializations in cocultured-primary neurons exemplifies the synaptogenic capability of CAMs (Scheiffele et al., 2000). Conversely, the reverse applies to neurexin—its presynaptic expression is sufficient to induce the formation of postsynaptic compartments (Graf et al., 2004). Despite their distinct role in synaptogenesis, the deletion of individual synaptic organizers does not adversely disrupt neurological development. Given the frequent presence of alternative genes, promoters, splice site in CAM coding sequences (Chih et al., 2006; Schor et al., 2013; Treutlein et al., 2014; Li et al., 2020), and preferential binding partners, the possible permutations of the interactions (Fowler et al., 2017; Nozawa et al., 2022), revealing the precise nanoscopic architecture of CAM interactions, is a monumental task. The importance of CAMs in neurological development can only be addressed with the resolution of the relative contribution of each type of CAMs in neurological development and function.

Presynaptic proteins that support the function of CAMs and synaptic organization complicate the process of synaptogenesis and specification. Liprin was identified as an interacting partner and regulator of the localization of the adhesion molecule leukocyte common antigen-related receptor protein tyrosine phosphatase (LAR-RPTP) (Serra-Pagès et al., 1995). As LAR-RPTPs were required for the synaptogenic function of presynaptic neurexin (Han et al., 2020) and postsynaptic Slit- and Trk-like proteins (Slitrks) (Yim et al., 2013), presynaptic Liprin can modulate synapse specification. The vertebrate Liprin family of proteins consists of four different isoforms of Liprin-α (α1–4), two Liprin-β (β1, 2), and a single KazrinE (Sakamoto et al., 2012). In mammals, liprin-α1 is ubiquitously expressed throughout the body, while Liprin-α2 and 3 are predominant in the central nervous system (CNS). Rare *de novo* missense mutations and chromosomal rearrangement that disrupt the intronic sequence of the Liprin-α1 coding gene PPFIA1 were found in autism proband and patients (Schluth-Bolard et al., 2013; Iossifov et al., 2014).

Liprin-α is also involved in the interaction and recruitment of several components of the presynaptic vesicle release machinery, such as the calcium/calmodulin-dependent protein kinase II (CaMKII), calcium/calmodulin-dependent serine protein kinase (CASK), and regulating synaptic membrane exocytosis protein 1 (RIM1) (Spangler and Hoogenraad, 2007; Spangler et al., 2013), to facilitate synaptic transmission. Interestingly, activity-dependent phosphorylation of neurexin by CASK destabilizes the CASK-Liprin-Neurexin complex, leading to dissociation and an increase in turnover of neurexin (LaConte et al., 2016). Consistent with the observation, mutations in the *Caenorhabditis elegans* homolog of Liprin-α syd-2 caused the a decrease in active site electron density, mislocation of synaptobrevin-GFP labeling in the active zone, and an increase in the overall size of the presynaptic active site, without affecting synaptic density (Zhen and Jin, 1999). A similar observation was made in the mammalian system—the depletion of Liprin-α2/3 in mice results in the disruption of the active zone ultrastructure, synaptic vesicle tethering, and vesicle exocytosis (Spangler et al., 2013; Wong et al., 2018). Liprin-α is further involved in postsynaptic Slitrk6 and neuroligin 2 (NLGN2)-mediated presynaptic differentiation in cultured rat hippocampal neuron culture (Han et al., 2018), likely via its function as a presynaptic anchor and organizer.

### Synaptic vesicle priming

Upon targeted interactions between CAMs, there is a rapid and bulk recruitment of preassembled active zone proteins in the form of piccolo-bassoon transport vesicles (PTVs) and synaptic vesicle protein transport vesicles (STVs) (Sabo and McAllister, 2003; Shapira et al., 2003; Sabo et al., 2006; Tao-Cheng, 2007). These are thought to be readily integrated into the nascent presynaptic structure, and form the basis for further defined recruitment and localization of the functional subunits of the presynaptic release machinery. Regulating synaptic membrane exocytosis (RIM), RIM-binding protein (RIM-BP), protein-rich in the amino acids glutamic acid (E), leucine (L), lysine (K), and serine (S) (ELKS), and Liprins interact with each other to form a large protein network that recruits the necessary molecular components for effective vesicle exocytosis (Südhof, 2012). Rare inherited mutations in RIM and *de novo* RIM-BP variants have been identified in the human ASD population (Bucan et al., 2009; Iossifov et al., 2012; Dong et al., 2014). The localization of voltage-gated calcium channels (VGCC) in the presynaptic compartment is directly dependent on the interaction between their cytoplasmic domain and the PDZ domain of RIM and RIM-BP (Kaeser et al., 2011), while the interaction of RIM with Munc13 places Munc13 near the soluble N-ethylmaleimide sensitive factor attachment protein receptor (SNARE) machinery for the efficient coupling of vesicle priming and docking events (Betz et al., 2001; Dulubova et al., 2005; Ma et al., 2011). Indeed, the deletion of RIM or the RIM-BP results in the loss of presynaptic Ca^2+^ channels, reduced synaptic release probability in the immediate proximity of the presynaptic active zone, and impairment in activity-dependent neurotransmitter release (Kaeser et al., 2011; Liu et al., 2011; Zarebidaki et al., 2020). When combined with mutations in presynaptic factors associated with RIM or RIM-BP function, basic presynaptic homeostatic plasticity fails in *Drosophila* (Genç et al., 2020), suggesting a potential convergence of seemingly unrelated autism risk genes to the susceptibility of disturbed presynaptic function.

Distinct from the neurotransmitter-containing synaptic vesicles, large dense core vesicle (LDCV) exocytosis is regulated by the Ca^2+^-dependent secretion activator (CAPS), a two-member multidomain protein (Berwin et al., 1998; Renden et al., 2001). However, the enrichment of CAPS proteins in the neuron presynaptic compartments where there is very little LDCV (Sadakata et al., 2006) raised questions surrounding their possible involvement in synaptic vesicle exocytosis. Subsequent studies revealed their fundamental importance in priming synaptic vesicles for fusion and neurotransmitter release. CAPS-1 ablation in mouse hippocampal neurons drastically reduced the readily releasable pool (RRP) size and halved the evoked excitatory postsynaptic current (EPSC) amplitude, despite comparable release probability during RRP release and EPSC kinetics (Jockusch et al., 2007). Furthermore, the double knockout of CAPS renders a significant proportion of the analyzed neurons (39%) incapable of eliciting an EPSC response even with hypertonic buffer treatment. The functional defects observed are due to the loss of docked vesicles with CAPS-1 knockout (Shinoda et al., 2016). CAPS mutations are further linked to the disrupted release of neuropeptides involved in the modulation of social behavior (Fujima et al., 2021). This is in line with the observation that ASD patients have lower blood oxytocin concentration (Zhang et al., 2016), and the oxytocin-treatment-dependent improvement in social skills is only present in individuals with lowered oxytocin levels (Parker et al., 2017). Nonetheless, there have been mixed results in clinical trials involving oxytocin-related treatments, and the efficacy of oxytocin in enhancing the social function of ASD patients remains unclear. Although CAPS mutations are rare in human autism patients (Sadakata et al., 2007; Bonora et al., 2014), likely due to CAPS’ functional importance in the nervous system, gene mutations directly impacting the priming function of CAPS-1/2 would be easily translated into behavioral changes.

### Synaptic vesicle exocytosis

The process of synaptic vesicle recruitment, priming, fusion, and recycling during chemical neurotransmission has been extensively examined (Okamoto and Südhof, 1997; Rizo and Rosenmund, 2008; Südhof, 2013; Imig et al., 2014; Chanaday et al., 2019). Due to the ubiquitous nature of Ca^2+^ in excitable cell function, function-modifying mutations in the VGCC have been identified in several neurological and neuromuscular conditions, including ASD (Bidaud et al., 2006; Lu et al., 2012). In the neuron, activity-dependent activation of the VGCC and influx of Ca^2+^ in the presynaptic terminal is a critical step in the release of neurotransmitters. The formation and assembly of the SNARE complex are heavily dependent on the presence of Ca^2+^ (Chen et al., 1999) whereby the forebrain-specific ablation of calcium voltage-gated channel subunit α1 A (CACNA1A) results in deficits in a variety of cognitive functions related to learning and memory and circadian rhythms (Mallmann et al., 2013). Depending on the site of impact, mutations in calcium voltage-gated channel subunit α (CACNA) which encodes for the main pore-forming subunit of the VGCC likely have an impact on the activation profile and kinetics of VGCC function.

Synaptic vesicles containing neurotransmitters can translocate between presynaptic terminals (Staras et al., 2010), and the vesicle distribution can be affected by synapsin (SYN) and β-catenin (Bamji et al., 2003; Pechstein et al., 2020), which sequester mobile vesicles. SYN is a family of evolutionarily conserved presynaptic proteins (Candiani et al., 2010) that plays a critical role in the structural and functional organization of the presynaptic terminal. Particularly, it reversibly tethers synaptic vesicles to the actin cytoskeleton which is crucial for the establishment of the vesicle pool for efficient neurotransmission (Baldelli et al., 2007; Cesca et al., 2010). Nonsense (Q555X) and missense (A550T and T567A) mutations, and maternally-inherited frameshift (A94fs199X) and missense (Y236S and G464) mutations have been identified in SYN1 and SYN2, respectively (Fassio et al., 2011; Corradi et al., 2014). Defects in directed targeting of the A550T and T567A SYN1 mutants, the lack of expression of the nonsense A94fs199X variant of SYN2, and the ablation of SYN2 function with Y236S and G464R revealed the importance of the SYN family activity in the regulation of synaptic function. The onset of SYN expression coincides with neuronal differentiation and peaks during synaptogenesis (Haas and DeGennaro, 1988; Melloni and Degennaro, 1994). Beyond this, SYN knockout in mice results in a differential impact on the populations of synaptic vesicles within the excitatory and inhibitor terminals (Gitler, 2004; Chiappalone et al., 2009), alongside epileptic phenotypes and ASD-like behaviors (Li et al., 1995).

It is recognized that spontaneous neurotransmitter release is important in the regulation of presynaptic maturation in the developing nervous system (Choi et al., 2014), as well as the regulation of postsynaptic receptor clustering and synaptic strength (Saitoe et al., 2001). Spontaneous release is dependent on the function of the putative presynaptic calcium sensors synaptotagmin (SYT), double C2-like domain-containing protein alpha (DOC2α), and double C2-like domain-containing protein beta (DOC2β) (Xu et al., 2009; Groffen et al., 2010), which differs in their cell-type specific expression profile (Courtney et al., 2018), sensitivity toward Ca^2+^, kinetics, and preference for phospholipid binding (Kojima et al., 1996; Groffen et al., 2006). Calcium binding changes the conformation of C2 domains, allowing them to insert into, buckle, and bring the presynaptic membrane closer to the synaptic vesicle to promote membrane fusion and neurotransmitter release (Martens et al., 2007). Mutations of SYT1 clustered mainly within the C2B domain which affected exocytosis rates following sustained action potential stimulation (Baker et al., 2015, 2018). SYT with M303K, D304G, and D366E mutations specifically failed to localize or relocalize to the presynaptic terminals following exocytosis. Comparatively, the DOC2A M225I variant likely affects its interaction with Munc13 (Kumar et al., 2009) and consequently SNARE-dependent fusion of synaptic vesicles. Hence, phenotypes associated with autism-associated SYT and DOC2 mutations originate from the disturbed neuronal transmission.

The importance of the SNARE complex in vesicle exocytosis cannot be overstated. Spontaneous membrane fusion in living organisms is energetically unfavorable—a large amount of directed force is required to overcome the repulsive forces between the lipid structures and lateral tension on the membrane surface (Chernomordik et al., 1987; Kozlovsky et al., 2002). Cooperative interactions between the vesicle SNARE (v-SNARE) synaptobrevin, and target-localized SNAREs (t-SNAREs) syntaxin-1 and synaptosomal-Associated Protein, 25kDa (SNAP-25) complex provide sufficient mechanical force for the fusion of opposing bilayers (McNew et al., 2000). Amisyn is a brain-enriched protein with a tomosyn- and synaptobrevin (VAMP)-liked coiled-coil-forming domain that competes with synaptobrevin-2 (VAMP2) for the assembly of the SNARE complex and inhibition of SNARE-dependent vesicle priming (Kondratiuk et al., 2020). Amisyn-containing SNARE complexes are more thermally stable than conventional VAMP2-containing SNARE complexes (Scales et al., 2002), but fusion-incompetent due to the absence of a transmembrane anchor in Amisyn. Interestingly, amisyn or syntaxin binding protein 6 (STXBP6) knockout in mice does not lead to any behavioral abnormalities (Liu et al., 2021), suggesting that Amisyn is not critical for neuronal development but may be involved in the regulation of activity-dependent of synaptic vesicular release. To date, further information regarding the regulatory mechanism of Amisyn function is absent, with only speculations of the full implication of STXBP6 mutation in ASD.

### Retrieval of vesicle from plasma membrane and endocytosis

Following activity-dependent exocytosis, synaptic vesicles can be regenerated through the reuse of synaptic vesicles that have transiently docked and fused with the synaptic membrane (kiss-and-run fusion) (Alabi and Tsien, 2013), clathrin-mediated endocytosis of fully collapsed lipid structures from the plasma membrane, activity-dependent bulk endocytosis (Cheung et al., 2010) or fast endophilin-mediated endocytosis (Watanabe and Boucrot, 2017). Dual Specificity Tyrosine Phosphorylation Regulated Kinase 1A (DYRK1A) encodes a chromosome 21-associated proline-directed serine/threonine kinase with dual function in the regulation of gene transcription and clathrin-mediated endocytosis. Recurring DYRK1A haploinsufficiency has been observed in ASD and is associated with microcephaly, intellectual disability, and epileptic seizures (Earl et al., 2017).

At the early stages of neurodevelopment, DYRK1A phosphorylation of Lin-52 dREAM MuvB core complex component (LIN52) is required for the assembly of the dimerization partner (DP), retinoblastoma (RB)-like, E2F, and multi-vulval class B (MuvB) (DREAM) complex which coordinates cell cycle-dependent gene expression (Litovchick et al., 2011; Sadasivam and DeCaprio, 2013). Despite the presence of a nuclear localization sequence, the majority of the DYRK1A remained localized to the cytoplasm of neurons (Hämmerle et al., 2003; Martí et al., 2003). DYRK1A was detected in isolated latherin-coated vesicles and colocalized with latherin in mouse neurons. Mass spectrometry analysis revealed the phosphorylation of adaptins, dymain 1 (DYN1), amphiphysin 1 (AMPH1), and synaptojanin 1 (SYNJ1) by DYRKA1 which inhibits the onset of latherin-mediated endocytosis and promotes the dissociation of clathrin structures on vesicles (Murakami et al., 2012). While the autism-associated R205X and E239X truncations are linked to defects in dendritic growth and spine development in rodents (Dang et al., 2018), the normalization of DYRK1A expression postnatal is sufficient to ameliorate synaptic and the functional changes linked to altered synaptic plasticity (Duchon and Herault, 2016). Similarly, the multimodular intersectin 1 (ITSN1) has been shown to interact with endocytic proteins and control endocytosis in various cell types and organisms, with contradictory effects observed for mammalian synaptic vesicle endocytosis (Gubar et al., 2013).

Although synaptophysin (SYP) is the most abundant synaptic vesicle membrane protein, its function is enigmatic. The existing report suggests the diverse roles of SYP function, including synaptogenesis (Tarsa and Goda, 2002), and the biogenesis of synaptic vesicles (Leube et al., 1989; Cameron et al., 1991; Kwon and Chapman, 2011). Mutant mice lacking SYP are viable and does not exhibit changes in neuronal structure and behavior (McMahon et al., 1996). The lack of phenotypes is accounted for by the redundancy of function between the synaptophysin and synaptogyrin family of proteins (Raja et al., 2019). Interestingly, SYP knockout results in the mislocalization of VAMP2 with no impact on vesicle turnover, likely reflecting a defect in SYP-dependent VAMP2 retrieval during synaptic vesicle endocytosis (Gordon et al., 2011). As such, while SYP variants have been identified in a population of Vietnamese autistic patients (Tran et al., 2020) and subjects recruited by the Autism Sequencing Consortium (Satterstrom et al., 2020), their role in ASD is not known.

### Regulation of synaptic vesicle filling and synaptic vesicle pools

Regardless of the mechanism adopted for the replenishment of synaptic vesicles, empty vesicles need to be rapidly refilled in the anticipation of a new round of neuronal activity and neurotransmission. Partially filled synaptic vesicles have a lower release probability (Rost et al., 2015) and can affect signal transmission. Vacuolar H^+^-ATPase (vATPase) is first required to establish a proton gradient across the vesicle membrane before neurotransmitters can be efficiently loaded into the vesicle lumen through the established electrochemical gradient (Farsi et al., 2017). The precise molecular composition and arrangement of synaptic-vesicle-associated vATPase have not been fully resolved.

Unlike conventional ASD risk genes, the correlation of nuclear receptor coactivator (NCOA7) with autism pathogenesis was revealed through the multidimensional examination of shared co-expression relationships of previously identified autism candidate genes with normal neurodevelopmental processes (Mahfouz et al., 2015). Up to date, there is only a single recessive case of autism known to be due to mutation in nuclear receptor coactivator 7 (NCOA7) (Autism Sequencing Consortium et al., 2019), and very little is known about NCOA7 function in neuronal physiology. NCOA7 is widely expressed throughout all developmental time frames of the mouse brain and interacts with various cytosolic V1 subunits of the vATPase *in vivo*. The loss of NCOA7 results in the increase in the number of proximal neurites of cultured primary neurons and a reduction in inhibitory synapses in layer 2/3 of the somatosensory cortex, which is related to the impaired social behavior observed (Castroflorio et al., 2021). A closer examination of potential ASD risk factors linked to vATPase function could reveal novel mechanisms for the development of neurodevelopmental and neuropsychiatric disorders.

The Na^+^/H^+^ exchanger 6 and 9 (NHE6/9 or SLC9A6/9) has been identified as candidate gene of interest for attention deficit hyperactivity disorder (ADHD) (Lasky-Su et al., 2008) and ASD (Morrow et al., 2008). Nonsense mutations that result in a similar premature termination of the last transmembrane segment of NHE9 have been observed in the closely related NHE1 and NHE6, associated with lower cognitive ability and epilepsy (Cox et al., 1997; Gilfillan et al., 2008). The sensitivity of Na uptake by glutamatergic synaptic vesicles toward a low micromolar amount of 5-(N-ethyl-N-isopropyl)amiloride, an inhibitor of NHEs suggests the involvement of NHEs in the filling of synaptic vesicles (Goh et al., 2011). Furthermore, mutations in the secretory carrier-associated membrane protein 5 (SCAMP5), a synaptic vesicle enriched protein responsible for the trafficking and synaptic localization of NHE6, were reported in idiopathic ASD and autism-like neurodevelopmental disorder with the manifestation of epilepsy (Castermans et al., 2010; Hubert et al., 2020). The knockdown of SCAMP5 in rat hippocampal neurons mislocalized NHE6 and hyperacidified the synaptic vesicles within the neuron (Lee et al., 2021). Given the prominent endosomal function of NHE, a careful evaluation of the contribution of NHEs is warranted before any conclusion can be made regarding its role in ASD development.

## Discussion

ASD diagnosis and treatment remain difficult despite increasing research and clinical efforts to tackle the condition. Due to the essentiality of synaptic genes in proper neurological function, it is not surprising that many of the reported ASD risk genes were associated with alternate neuropsychiatric conditions (Zhu et al., 2014). However, varying neurological or non-neurological-related developmental trajectories can result in a similar cognitive outcome (Sala et al., 2020). Furthermore, patients with ASD often suffer from other comorbid conditions (Rosen et al., 2018) that may mask proper ASD symptoms, delay diagnosis (Mazefsky et al., 2012), and affect treatment efficacy (McDougle et al., 2003). There is currently no efficient method for identifying the causal factor and linking it directly to potential neurological defects in ASD patient, and neither is there a consensus on the optimal instrument for measuring the co-occurrence of other psychiatric disorders in ASD.

In addition to the above reported presynaptic targets, there was a pickup of mutations in alternate presynaptic factors with unknown significance in ASD. Individuals diagnosed with delayed development and amyotrophic lateral sclerosis carry UNC13A (coding for Munc13-1) (Engel et al., 2016; Tan et al., 2020), syntaxin-binding protein (STXBP1, coding for Munc18-1) in individuals with intellectual disorder and epilepsy (Hamdan et al., 2009; Carvill et al., 2014), and dynamin 1 (DMN1) in epilepsy (Appenzeller et al., 2014). Despite their equal importance in presynaptic function and neurotransmission, the restricted clinical outcome and lack of association with autism are surprising. We recognize that a multifactorial model may be a better representative of the development of ASD (Guo et al., 2018). Even among closely related genes, the skewed prevalence of mutations in specific targets is particularly perplexing. Although current genetic sequencing methods have been efficient in covering a significant proportion of the protein-coding region of the human genome, annotations of GC-rich sequences (Kim et al., 2022), the consensus in heterogenous locus, low-level genetic mosaics due to somatic *de novo* mutations (Rodríguez-Santiago et al., 2010), and gender biases have not been adequately examined (Jacquemont et al., 2014). Unless validated, caution is necessary for implicating single instances of mutations with ASD pathogenesis.

Experimental animal models are actively employed to understand the biological relevance of human mutations in neurological functions. Genetically modified rodent models have provided invaluable insights into the precise role of several presynaptic factors in specific neurological functions (Annamneedi et al., 2021; Castroflorio et al., 2021; Mitsogiannis et al., 2021). Nonetheless, the generation of animal models that would fully recapitulate the autistic human behavioral phenotype is difficult, if not currently impossible. ASD diagnosis is based purely on the characteristic behavioral defects in social interactions, communication, and motor stereotypes, which cannot be recapitulated satisfactorily with stereotypic rodent behavior and existing behavioral tests (Silverman et al., 2010). Although behavioral studies are often supplemented with biochemical and electrophysiological assays to provide a comprehensive understanding for the analysis of target function, inherent differences in the structural and molecular organization of synapses between humans and rodents (Testa-Silva, 2010; Testa-Silva et al., 2014; Koopmans et al., 2018; Wildenberg et al., 2021) reduce the relevance of phenotypes observed to human conditions. Cell reprogramming methods combined with pre-existing animal models, such as the human neuron xenograft models (Real et al., 2018; Linaro et al., 2019), and advanced analysis tools are valuable for understanding the role of presynaptic genes in actual human neurological function.

While ASD patients often suffer from the cognitive impact of irreversible neurodevelopmental defects (Xiao et al., 2014; Kumar et al., 2019), the sensitivity of human behavior and cognitive function to regulation from multiple molecular levels (Ganguly and Poo, 2013; Juárez-Portilla et al., 2018) provides opportunities to reverse autistic symptoms even later in life. Synaptic remodeling continues through adulthood after the conclusion of the critical stages of neurogenesis, cell migration, and maturation in the nervous system (Zito and Svoboda, 2002; Kelsch et al., 2008). Interestingly, the adult restoration of synaptic protein expression can reverse part of the autism-like phenotypes observed in mice (Mei et al., 2016). Furthermore, the neuronal nicotinic acetylcholine receptor agonists Nefiracetam and PHA 543613 developed for Alzheimer’s disease treatment can reverse synaptic defects observed in an induced human pluripotent stem cell model of MECP2 knockout cortical organoid (Trujillo et al., 2021). Although FDA-approved modifiers of synaptic function such as memantine and ketamine have met with a certain level of success in clinical use (Blanco-Silvente et al., 2018; Nuñez et al., 2020), their effect is mediocre likely due to the difference in etiology between patients. There are also no existing drugs targeting specifically presynaptic proteins and functions. Further considerations are essential for effective novel presynapse-targeting therapeutics development against the greatly heterogenous ASD etiology and symptoms.

## Author contributions

XY and SJ: conceptualization. XY, YL, CP, HP, and SJ: writing—original draft preparation. XY, WC, HP, and SJ: writing—review and editing. SJ: supervision. HP and SJ: funding acquisition and critical revision of manuscript. All authors have read and agreed to the published version of the manuscript.
